# Primary healthcare-friendly prostate cancer prediction model using routine clinical parameters: a multicenter study

**DOI:** 10.3389/fonc.2026.1787090

**Published:** 2026-03-31

**Authors:** Ming Chen, Tingting Li, ShuPing Yang, You Zhou, Hanzong Lin, Jinxin Lan, Xiaoqian Zhang, Xiao Yang, Jiguang Zhou, Guorong Lyu

**Affiliations:** 1 Department of Ultrasound, Second Affiliated Hospital of Fujian Medical University, Quanzhou, Fujian, China; 2 Department of Ultrasound, Zhangzhou Affiliated Hospital of Fujian Medical University, Zhangzhou, Fujian, China; 3 Department of Ultrasound, Zhongshan Hospital, Fudan University (Xiamen Branch), Xiamen, Fujian, China; 4 Department of Ultrasound, Peking Union Medical College Hospital, Chinese Academy of Medical Sciences and Peking Union Medical College, Beijing, China; 5 Department of Statistics Room, Zhangzhou Affiliated Hospital of Fujian Medical University, Zhangzhou, Fujian, China

**Keywords:** external validation, nomogram, primary healthcare, prostate cancer, PSA

## Abstract

**Objective:**

This study aimed to develop and validate a nomogram model integrating routinely available parameters, making it applicable to primary healthcare settings for optimizing prostate cancer (PCa) risk stratification in patients with elevated prostate-specific antigen (PSA) levels, aiming to reduce unnecessary biopsies.

**Methods:**

This study included a retrospective cohort of 2, 844 patients who underwent prostate biopsy (885 malignant and 1, 959 benign cases), who were randomly allocated to a training set and an internal validation set in a 7:3 ratio. Independent predictive factors were selected through univariate analyses and multivariate logistic regression analyses, and a nomogram was constructed. The model’s performance was evaluated using Receiver Operating Characteristic (ROC) curve analysis, precision-recall (PR) curves, calibration curve, and decision curve analysis (DCA). Further validation was conducted using an external independent dataset (*n* = 281; 93 malignant and 188 benign cases) to assess the model’s generalizability.

**Results:**

The nomogram model incorporated prostate volume, total PSA (tPSA), free-to-total PSA ratio (f/t PSA), and age, demonstrating discriminative performance with AUC values of 0.816 (95% CI: 0.796–0.836; training set), 0.833 (95% CI: 0.802–0.862; internal validation set), and 0.776 (95% CI: 0.720–0.832; external validation set). Its clinically acceptable performance outperformed that of individual parameters in the training set (all p-values <0.001 by Delong’s test). The ROC curve and PR curve both demonstrated the robust predictive performance of the prediction model across all three study cohorts. The calibration curve showed strong agreement between the predicted probability and actual risk. DCA confirmed clinical net benefit across a wide range of risk thresholds.

**Conclusion:**

This nomogram provides a non-invasive, cost-effective, individualized PCa risk assessment tool for Chinese patients with elevated PSA levels. Its generalizability was confirmed through external validation, demonstrating effectiveness in optimizing biopsy decisions and suitability for primary healthcare settings, thereby reducing healthcare burden.

## 1 Introduction

Prostate cancer is the second most common malignancy in men worldwide, and timely screening and intervention can significantly improve survival rates (1). The implementation of noninvasive prostate cancer screening in primary healthcare settings could enhance screening accessibility, facilitate early detection of high-risk cases, and reduce diagnostic delays in resource-limited regions.

Although PSA testing has high sensitivity in prostate cancer screening, its specificity is relatively low, leading to numerous false-positive results and consequently causing overdiagnosis and unnecessary biopsies (2, 3). Studies have shown that while PSA testing can reduce mortality in some cases, its widespread use also brings about economic burden and psychological stress (3, 4). In order to improve the accuracy of prostate cancer screening, researchers are exploring other biomarkers and methods to enhance the specificity of PSA testing (5). For example, studies have found that a multi-biomarker urine sensor (6), combined with artificial intelligence analysis, is considered a promising precision screening strategy that can significantly improve screening accuracy (7). Additionally, personalized PSA screening strategies are also being researched. By integrating genetic information with PSA testing (8), personalized screening can reduce unnecessary PSA tests and overdiagnosis without compromising mortality reduction (9, 10). This method adjusts the PSA threshold to make screening more precise, thereby reducing unnecessary biopsies (11). However, these approaches all require specialized equipment and corresponding personalized screening data (12), and many studies still show unclear results (13), limiting their widespread application.

Therefore, establishing a simple yet accurate prostate cancer (PCa) prediction model to avoid unnecessary biopsy procedures is of crucial importance for prostate cancer screening and diagnosis, especially in primary healthcare settings. This study aims to establish a nomogram prediction model by selecting the most common clinical factors influencing prostate biopsy outcomes, with the hope that the model will accurately predict the risk of prostate cancer and be applied in primary healthcare institutions, providing essential guidance and support for urologists in formulating diagnostic and treatment plans.

## 2 Materials and methods

### 2.1 Study population and ethical approval

This study is a multi-center retrospective cohort study, with the primary cohort data sourced from the Department of Ultrasound at Zhangzhou Hospital in Fujian Province, covering patients who underwent ultrasound-guided transrectal or transperineal prostate biopsy between October 2007 and December 2024. An independent external validation dataset was obtained from a collaborating hospital (the Second Affiliated Hospital of Fujian Medical University) using the same inclusion and exclusion criteria. This study received ethical approval from the Institutional Review Boards of both participating institutions: Zhangzhou Hospital of Fujian Province (Approval No. 2025LWB624) and the Second Affiliated Hospital of Fujian Medical University (protocol code 233). The requirement for patient informed consent was waived. Data collection and processing strictly adhered to the principles of the Helsinki Declaration and the hospital’s privacy protection policies, with all patient information stored and analyzed in an anonymized form.

### 2.2 Study design

#### 2.2.1 Inclusion criteria

Ultrasound-guided transrectal or transperineal biopsy of patients with prostate cancer or benign lesions confirmed by pathology.

#### 2.2.2 Exclusion criteria:

​​ (1) Lack of key clinical parameters​​ (e.g., prostate volume measured by preoperative TRUS or preoperative PSA levels). ​​(2) ​​Recent use (within 6 months) of 5α-reductase inhibitors or androgen deprivation therapy (ADT).​​ ​​(3) Prior prostate-directed therapies​(surgery/radiotherapy/chemotherapy). ​​(4) Evidence of distant metastasis.​ (5) Acute prostatitis or documented urinary tract infection within 4 weeks prior to PSA measurement (may cause falsely elevated PSA); (6) Urinary catheterization within 48 hours before biopsy; (7) Use of 5α-reductase inhibitors (e.g., finasteride, dutasteride), which can reduce PSA by approximately 50%; (8) Recent (within 4 weeks) other invasive urological procedures.

**Figure 1:**
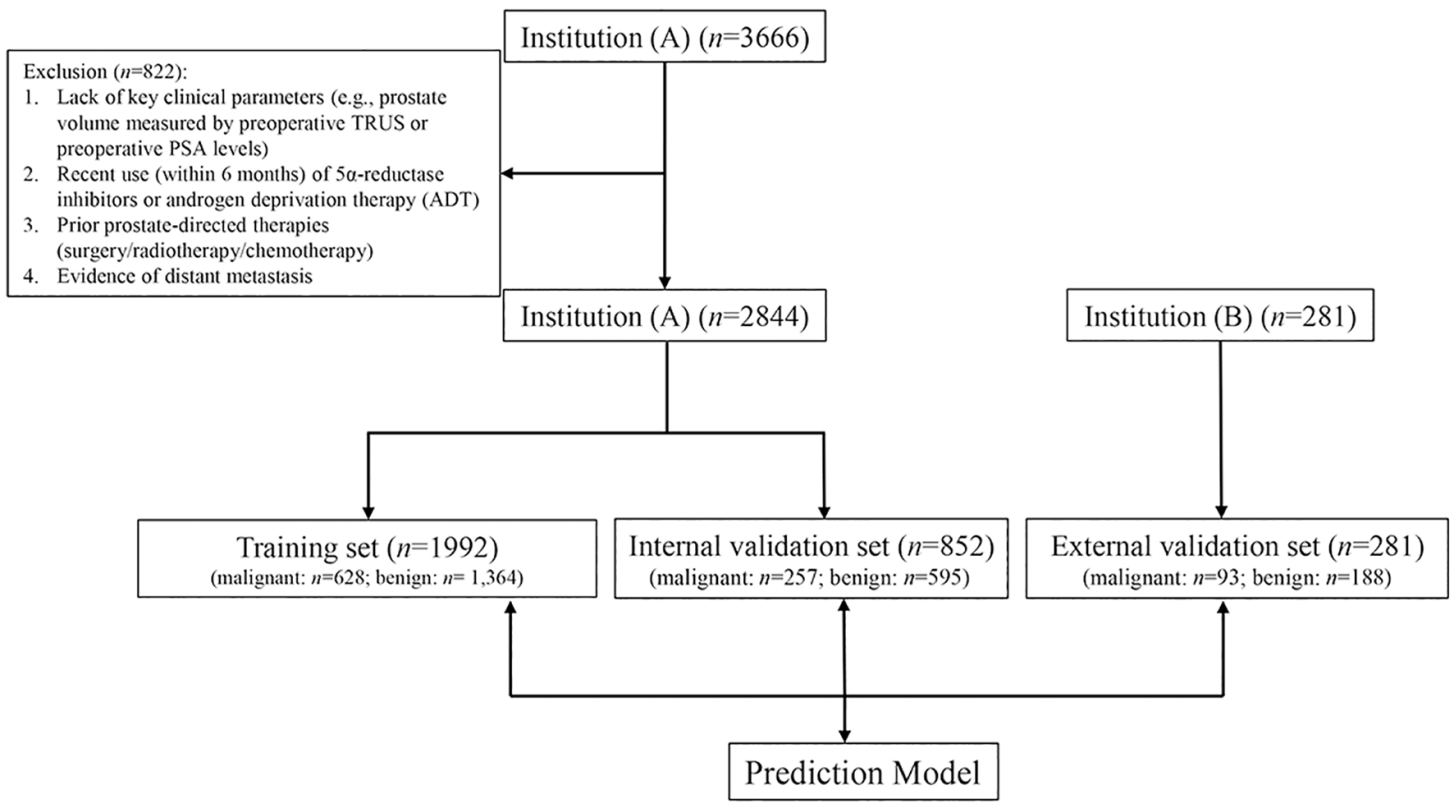
Flow chart of the study.

The primary cohort included a total of 2, 844 patients (malignant group: *n* = 885, 31.1%; benign group: *n* = 1, 959, 68.9%), who were randomly divided into a training set and an internal validation set at a 7:3 ratio. The external cohort consisted of 281 patients (malignant group: n=93, 33.1%; benign group: n=188, 66.9%) and served as the external validation set. The study flowchart is presented in **Figure 1**.

### 2.3 Data collection and preprocessing

#### 2.3.1 Clinical data extraction

The following data were extracted from the hospital’s electronic medical records (EMR): Demographic characteristics: age, medical history (including comorbidities such as hypertension/diabetes and lower urinary tract symptoms [LUTS] such as hematuria/dysuria/urinary frequency); Laboratory parameters: total PSA (tPSA), free PSA (fPSA), free-to-total PSA ratio (f/t PSA), prostate-specific antigen density (PSAD, PSAD = PSA value/prostate volume);Imaging parameters: prostate volume measured by transrectal ultrasound (calculated using the formula: 0.52 × length × width × height, unit: mL). For both the primary and external validation cohorts, digital rectal examination (DRE) and catheterization were prohibited within 72 hours before PSA testing.

#### 2.3.2 Data quality control

Data quality control measures were implemented throughout the study, including manual validation of outliers (e.g., PSA outliers) to identify and exclude potential testing or data entry errors. Missing data were addressed by excluding cases with incomplete key variables, particularly when clinical parameters (such as family history) were missing in more than 5% of cases. Comprehensive quality assurance protocols ensured standardized data collection procedures, strictly controlled biopsy protocols performed by physicians with at least five years of interventional ultrasound experience, and rigorous statistical review conducted by professional statisticians to validate analytical processes and maintain methodological integrity.

### 2.4 Development and validation of the model

Independent influencing factors for the predictive model were screened through univariate analysis and multivariate logistic regression analysis. Based on the training set data, variables selected by univariate analysis (p<0.05) were included in the stepwise multivariate logistic regression. Multicollinearity was assessed by calculating the variance inflation factor (VIF) within the full 7-variable candidate model, and variables with VIF > 10 were excluded. The overall model performance was evaluated using the Hosmer-Lemeshow goodness-of-fit test. The prediction model was visualized by constructing a nomogram, enhancing its clinical utility for practical application. The discriminative ability of the model was assessed by plotting the receiver operating characteristic (ROC) curve and calculating the area under the curve (AUC) along with its 95% confidence interval. The precision-recall (PR) curve analyzed model performance under different classification thresholds. Calibration validation was performed using the Bootstrap method with 1, 000 resamples, and a calibration curve was plotted to evaluate model fit. Additionally, decision curve analysis (DCA) was employed to compare the net benefit values of the “biopsy-all” and “biopsy-none” strategies at different threshold probabilities, thereby validating the clinical utility of the model.

### 2.5 Prostate biopsy procedure

All patients underwent ultrasound-guided transrectal or transperineal biopsy. Preoperative preparations included discontinuing anticoagulant medications 3 days prior to the procedure, a liquid diet the day before, and bowel preparation. The biopsy procedure followed a systematic approach with a 12-core template, taking samples from the bilateral base, midsection, and apex (2 cores from each area). Postoperative care involved applying pressure to stop bleeding for 20 minutes and monitoring vital signs for 2 hours.

### 2.6 Statistical analysis

Statistical analyses were performed using R software (version 4.5.1) and SPSS (version 31.0). The normality of continuous variables was assessed using the Kolmogorov-Smirnov test, with logarithmically transformed applied to non-normally distributed data prior to analysis. Normally distributed continuous variables were presented as mean ± standard deviation (SD) and compared using independent samples t-tests; non-normally distributed continuous variables were presented as median [interquartile range (IQR)] and compared using the Mann–Whitney U test. Normality was assessed using the Kolmogorov–Smirnov (KS) test. Categorical variables were described as frequencies (percentages), with group comparisons performed using chi-square tests or Fisher’s exact tests. The significance threshold was set at p < 0.05 (two-tailed). Logistic regression analysis was performed using the “glm” function in R software, and the “rms” package was employed to generate nomograms.

## 3 Results

### 3.1 Comparison of baseline characteristics

This study enrolled a total of 2, 844 patients who underwent ultrasound-guided transrectal or transperineal prostate biopsy in the primary cohort, with 885 cases classified as malignant and 1, 959 as benign. Baseline characteristic analysis revealed statistically significant differences (all p < 0.001) in the following features: The malignant group had significantly smaller prostate volumes than the benign group (48.02 [34.97–67.16] mL vs. 70.42 [51.28–95.24] mL; Mann–Whitney U test, p < 0.001). Both tPSA and fPSA levels were higher in the malignant group (26.16 [12.59–66.00] ng/mL vs. 12.78 [8.29–20.51] ng/mL and 3.07 [1.55–6.85] ng/mL vs. 1.96 [1.20–3.26] ng/mL, respectively; p < 0.001). The f/t PSA ratio was significantly lower in the malignant group (0.120 [0.082–0.160] vs. 0.150 [0.110–0.210]; p < 0.001). Both fPSAD and tPSAD were higher in the malignant group (0.060 [0.032–0.141] ng/mL² vs. 0.027 [0.018–0.042] ng/mL² and 0.536 [0.256–1.352] ng/mL² vs. 0.181 [0.122–0.295] ng/mL², respectively; p < 0.001). Additionally, patients in the malignant group were older (72.14 ± 8.42 years vs. 69.15 ± 8.39 years; t-test, p < 0.001). Among the 885 malignant cases, ISUP Grade 1 (Gleason ≤6) accounted for 181 cases (20.5%) and unclassifiable grading for 3 cases (0.3%). These cases were retained in model development as the nomogram functions as a pre-biopsy tool at which point Gleason grade is unknown; Grade 1 disease is not inherently clinically insignificant without pathological confirmation. Detailed baseline data analysis is presented in **Table 1**.

**Table 1:** Baseline Clinical Characteristics of the Study Cohort.

Variable	Malignant (n = 885)	Benign (n = 1,959)	P value
Age (years), mean ± SD	72.14 ± 8.42	69.15 ± 8.39	<0.001
Prostate volume (mL), median (Q1–Q3)	48.02 (34.97–67.16)	70.42 (51.28–95.24)	<0.001
tPSA (ng/mL), median (Q1–Q3)	26.16 (12.59–66.00)	12.78 (8.29–20.51)	<0.001
fPSA (ng/mL), median (Q1–Q3)	3.07 (1.55–6.85)	1.96 (1.20–3.26)	<0.001
f/tPSA ratio, median (Q1–Q3)	0.120 (0.082–0.160)	0.150 (0.110–0.210)	<0.001
tPSAD (ng/mL²), median (Q1–Q3)	0.536 (0.256–1.352)	0.181 (0.122–0.295)	<0.001
fPSAD (ng/mL²), median (Q1–Q3)	0.060 (0.032–0.141)	0.027 (0.018–0.042)	<0.001
Pathological grade (ISUP), n (%)			
Grade 1 (Gleason ≤ 6)	181 (20.5%)	–	–
Grade 2 (Gleason 3+4=7)	133 (15.0%)	–	–
Grade 3 (Gleason 4+3=7)	63 (7.1%)	–	–
Grade 4 (Gleason 8)	211 (23.8%)	–	–
Grade 5 (Gleason 9–10)	294 (33.2%)	–	–
Unknown/unclassified	3 (0.3%)	–	–
Clinically significant PCa (ISUP ≥ 2)	701 (79.2%)	–	–

SD, standard deviation; tPSA, total prostate-specific antigen; fPSA, free PSA; f/tPSA, free-to-total PSA ratio; tPSAD, total PSA density; fPSAD, free PSA density; ISUP, International Society of Urological Pathology; PCa, prostate cancer.

Age data follow a normal distribution (Kolmogorov–Smirnov test p > 0.05); continuous variables are presented as mean ± SD or median (Q1–Q3) as appropriate.

All P values calculated by independent samples t-test (age) or Mann–Whitney U test (others).

### 3.2 Univariate analysis and multivariate logistic regression analysis

As shown in **Table 2**, univariate analyses and multivariate logistic regression analyses were performed based on the training set data. Univariate analysis initially identified seven significant predictors (p < 0.05). However, collinearity diagnostics revealed that the variance inflation factor (VIF) values of fPSA (VIF = 34.27), fPSAD (VIF = 28.68), and tPSAD (VIF = 27.62) substantially exceeded the threshold of 10. Given the close correlation between fPSA and tPSA/f/t PSA, and considering that PSAD metrics are derived parameters calculated from PSA and prostate volume measurements, these variables exhibited high multicollinearity. To ensure model parsimony and stability, redundant derived variables with extreme VIF > 10 were excluded: fPSA is mathematically computed from tPSA and the f/tPSA ratio (both retained), while tPSAD and fPSAD are ratio-derived metrics from PSA and prostate volume (both already included as independent predictors). Note: the VIF values listed reflect the full 7-variable candidate model; the elevated VIF of the retained predictors (age, tPSA, f/tPSA) reflects their correlation with the now-excluded redundant variables, and is expected to be substantially reduced in the final 4-variable model. Subsequent stepwise multivariate logistic regression analysis ultimately identified prostate volume, tPSA, f/t PSA, and age as independent influencing factors for inclusion in the final predictive model. The regression coefficients and odds ratios (OR) demonstrated that: Prostate volume and f/t PSA were negatively associated with prostate cancer risk, indicating protective effects. Age and tPSA were positively associated with prostate cancer risk, serving as risk factors. The final logistic predictive model was derived as follows: ​​Logit (P) = -3.557 - 0.028 × Prostate Volume + 0.030 × tPSA - 2.215 × (f/t PSA) + 0.059 × Age​​.

**Table 2:** Univariate and Multivariate Logistic Regression Analysis for Prostate Cancer Prediction (Training Set, n = 1,990).

Variable	Univariate OR (95% CI)	Univariate P value	Multivariate OR (95% CI)	Multivariate P value	VIF
Age (years)	1.043 (1.033–1.054)	<0.001	1.060 (1.047–1.072)	<0.001	13.40
Prostate volume (mL)	0.978 (0.975–0.981)	<0.001	0.971 (0.968–0.975)	<0.001	7.21
tPSA (ng/mL)	1.030 (1.026–1.033)	<0.001	1.031 (1.027–1.035)	<0.001	31.88
fPSA (ng/mL)	1.176 (1.150–1.202)	<0.001	Excluded (VIF > 10)	–	34.27
f/tPSA ratio	0.002 (0.001–0.008)	<0.001	0.127 (0.036–0.447)	0.001	10.95
tPSAD (ng/mL²)	5.847 (4.737–7.216)	<0.001	Excluded (VIF > 10)	–	27.62
fPSAD (ng/mL²)	58225 (13112–258546)	<0.001	Excluded (VIF > 10)	–	28.68

OR, odds ratio; CI, confidence interval; VIF, variance inflation factor; tPSA, total PSA; fPSA, free PSA; f/tPSA, free-to-total PSA ratio; tPSAD, total PSA density; fPSAD, free PSA density.

Variables with VIF > 10 were excluded from the multivariate model due to multicollinearity. The final nomogram model incorporated prostate volume, tPSA, f/tPSA ratio, and age.

The Hosmer-Lemeshow test yielded a *p*-value > 0.05, indicating good model fit.

### 3.3 Construction of the nomogram prediction model

**Figure 2:**
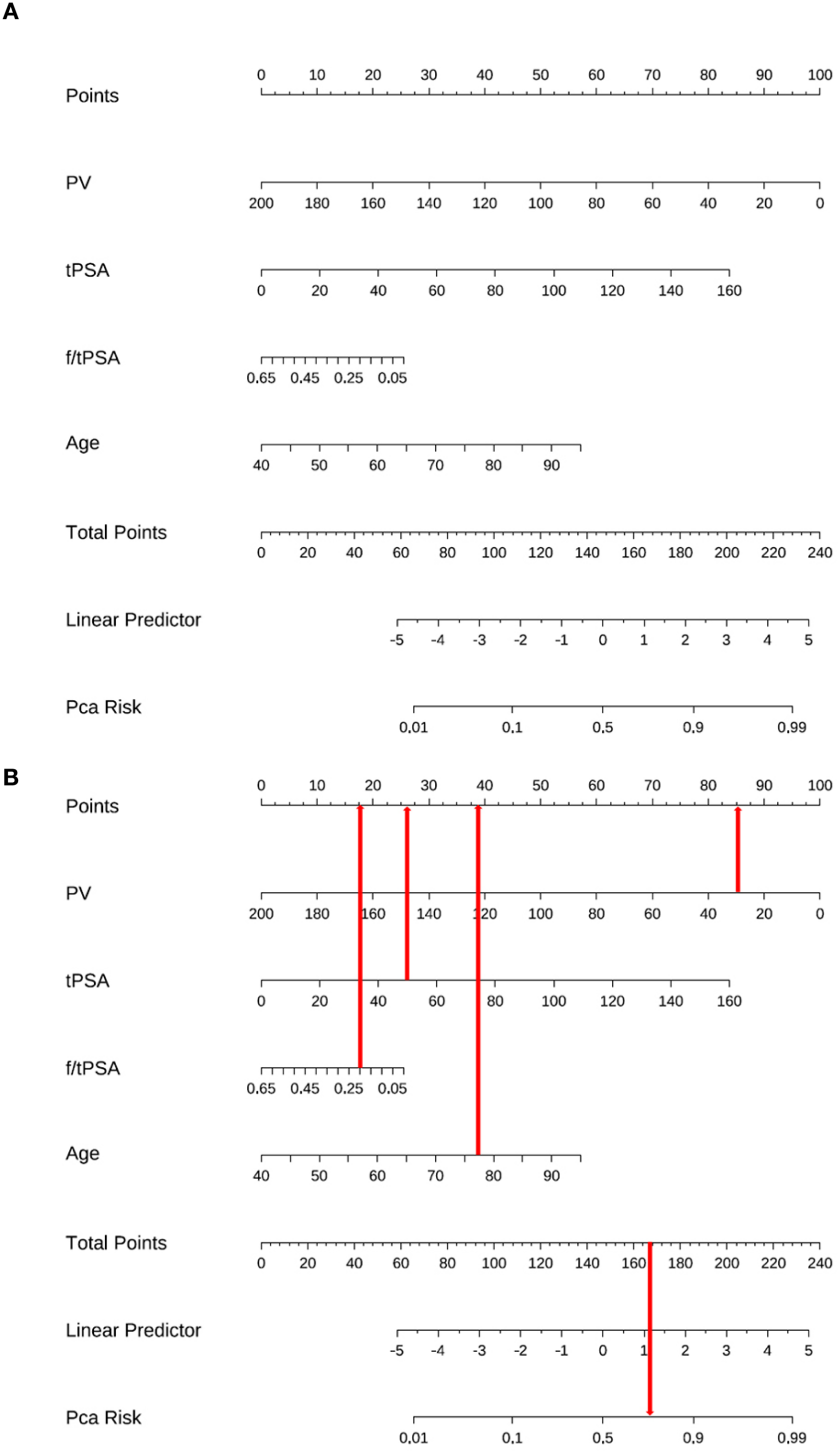
**(A)** The Nomogram based on the prediction model. **(B)** Case example: A 77-year-old male with prostate volume 29.98 mL (85 pts), tPSA 50.67 ng/mL (26 pts), f/t PSA 0.20 (17.5 pts), and age (38.5 pts) scored 167 points on our nomogram, predicting >70% prostate cancer risk. The pathological findings from ultrasound-guided transperineal prostate biopsy confirmed adenocarcinoma with a Gleason score of 5+5=10, validating the model’s accuracy.

Based on multiple influencing factors, including prostate volume, tPSA, f/t PSA, and age, a nomogram prediction model for prostate cancer was constructed using R software (**Figure 2**). This nomogram allows for the calculation of a total score by adding the scores corresponding to each predictor variable. The total score can then be used to calculate the predicted probability of prostate cancer. **Figure 2b** presents a representative case report.

### 3.4 Validation of the nomogram prediction model

**Figure 3:**
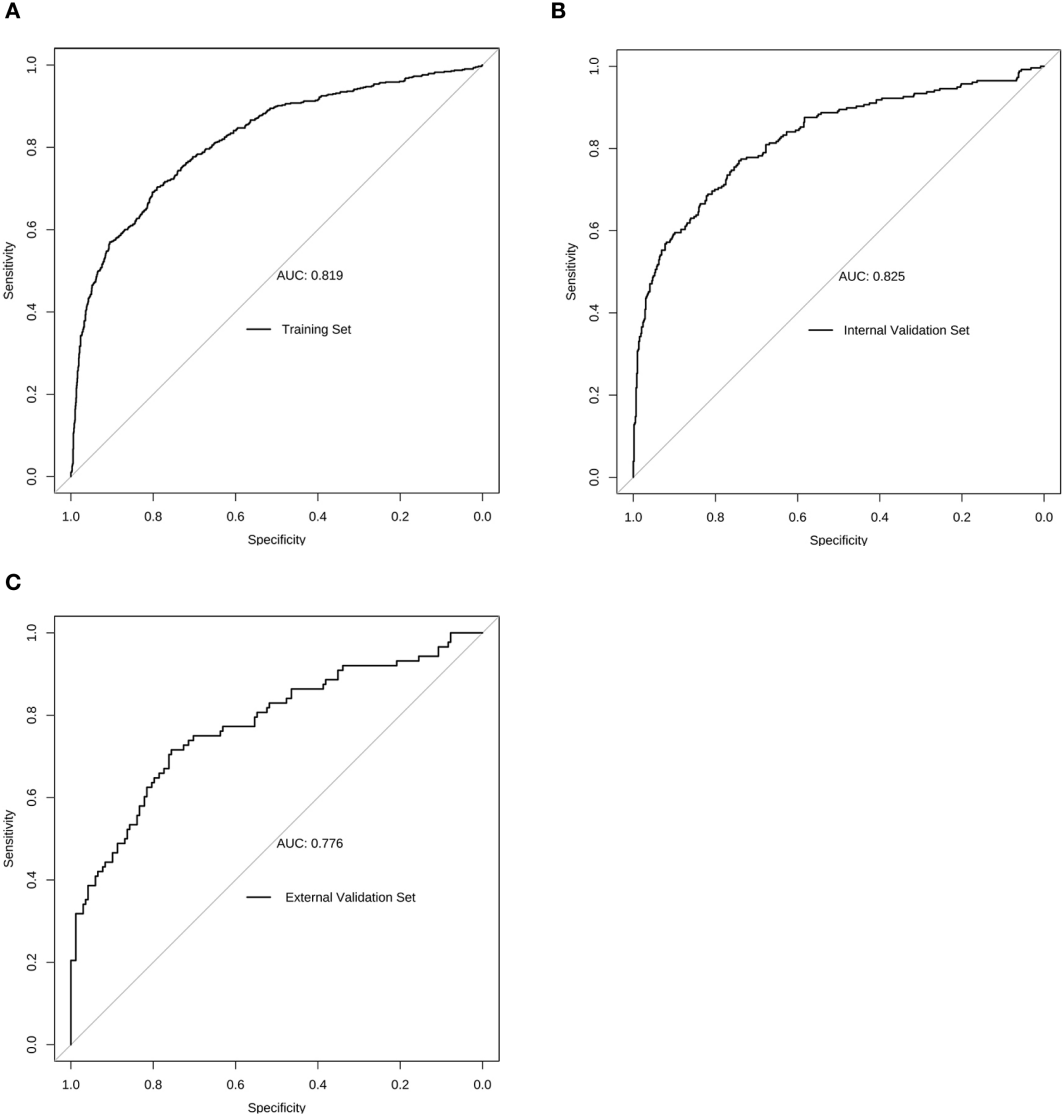
The Receiving Operating Characteristic (ROC) curves of the prediction model based on the **(A)** training set, **(B)** internal validation set, and **(C)** external validation set.

The DeLong test demonstrated that in the training cohort, the nomogram prediction model exhibited significantly superior diagnostic performance compared to individual parameters, with statistically significant differences (all p<0.001). Specifically, the model showed higher AUC values than single variables: prostate volume (0.816 vs 0.691), f/t PSA ratio (0.816 vs 0.638), age (0.816 vs 0.596), and tPSA (0.816 vs 0.712). This suggests that the multi-factor combined model has the best predictive ability for prostate cancer diagnosis, indicating that multi-factor combined assessment should be prioritized in clinical screening to improve screening accuracy and early detection rates. **Table 3** details the diagnostic performance of the nomogram prediction model across three study cohorts, including the training set, internal validation set, and external validation set. The ROC curves (**Figure 3**) demonstrate robust predictive performance of the nomogram model across all three study cohorts.

**Table 3:** Diagnostic Performance of the Nomogram Model and Individual Predictors.

Predictor / cohort	AUC (95% CI)	Sensitivity (%)	Specificity (%)	DeLong test vs nomogram (P)
Nomogram model				
Training set (n = 1,990)	0.816 (0.796–0.836)	72.0	76.6	Reference
Internal validation (n = 854)	0.833 (0.802–0.862)	73.9	77.7	Reference
External validation (n = 281)	0.776 (0.720–0.832)	71.6	75.6	Reference
Individual predictors (training set)				
Prostate volume	0.691 (0.665–0.717)	–	–	<0.001
tPSA	0.712 (0.686–0.738)	–	–	<0.001
f/tPSA ratio	0.638 (0.611–0.665)	–	–	<0.001
Age	0.596 (0.568–0.624)	–	–	<0.001

AUC, area under the receiver operating characteristic curve; CI, confidence interval.

AUC 95% CIs calculated by 1,000-bootstrap resampling. Optimal probability threshold (≈ 0.31) determined by the Youden index on the training set. DeLong test used to compare AUCs between the nomogram and each individual predictor.

tPSA, total PSA; f/tPSA, free-to-total PSA ratio.

External validation cohort statistics are derived from a separate multicenter dataset (n = 281); sensitivity and specificity for external validation cohort apply the same Youden-derived threshold.

**Figure 4:**
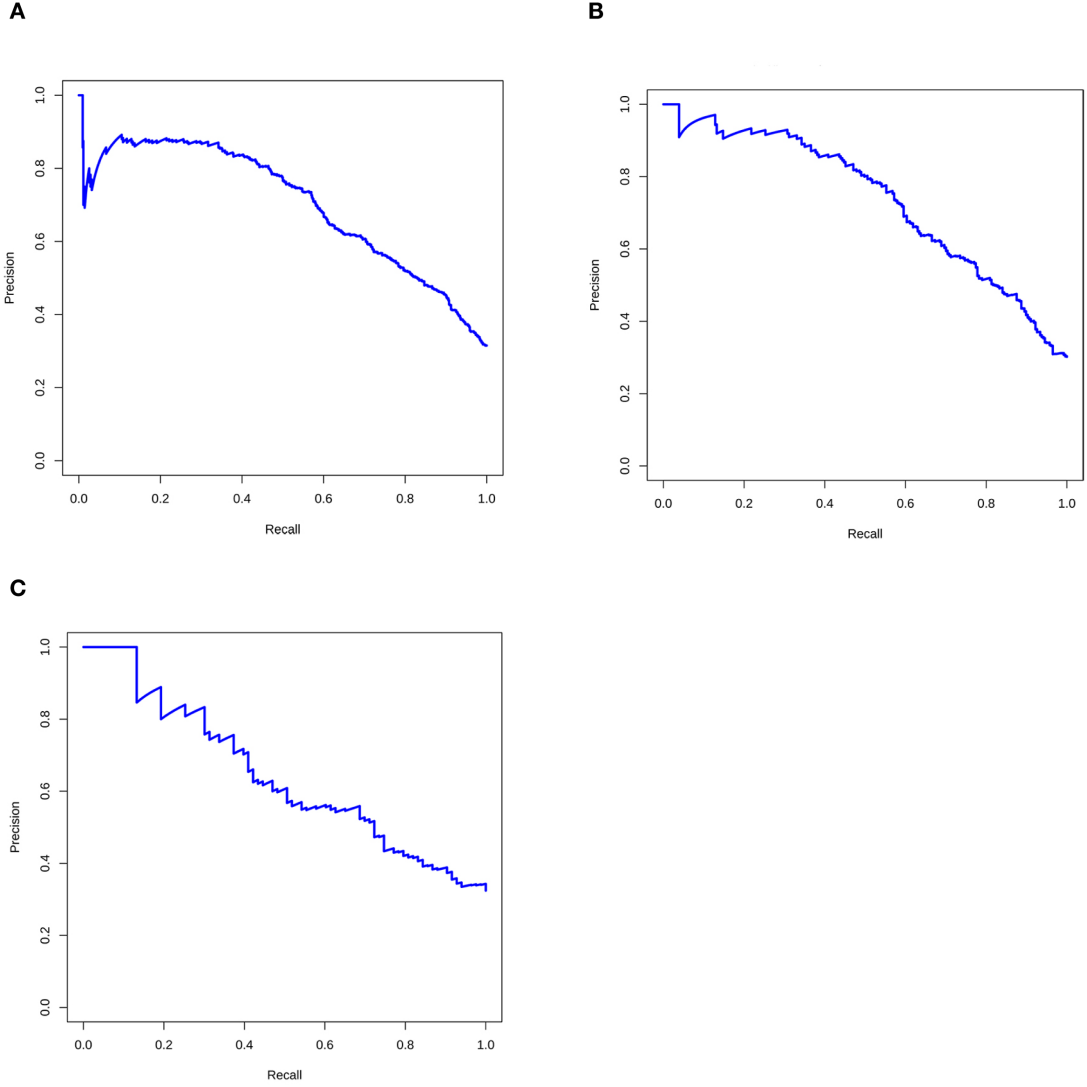
The Precision-Recall (PR) curves of the prediction model based on the **(A)** training set, **(B)** internal validation set, and **(C)** external validation set.

Given the imbalanced class distribution in this study (malignant cases accounted for 31.1% vs. 68.9% benign in the primary cohort, and 33.1% vs. 66.9% in the external validation cohort), we additionally employed Precision-Recall (PR) curves (**Figure 4**) to validate the predictive model’s performance. The results demonstrated consistently robust performance of the nomogram prediction model across all three study cohorts.

**Figure 5:**
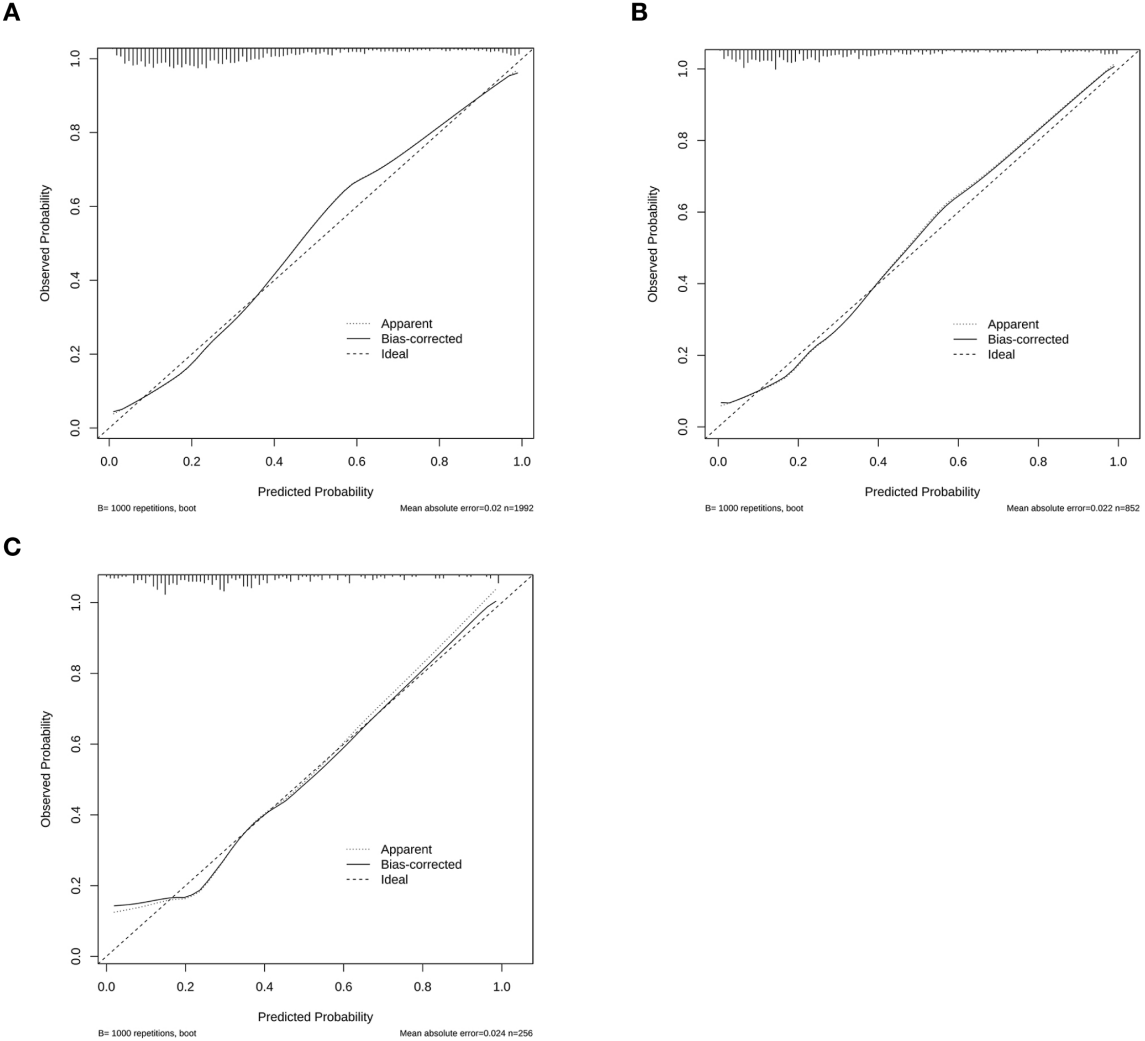
The Calibration curves of the prediction model in the **(A)** training set, **(B)** internal validation set, and **(C)** external validation set.

The calibration curve is presented in **Figure 5**. The dashed line (Apparent line) represents the initial model’s prediction, the solid line (Bias-corrected line) represents the prediction after bias correction, and the dotted line (Ideal line) represents the perfect prediction. The calibration curve shows that the Bias-corrected line closely follows the Ideal line, indicating that the corrected model has high predictive consistency and accuracy.

**Figure 6:**
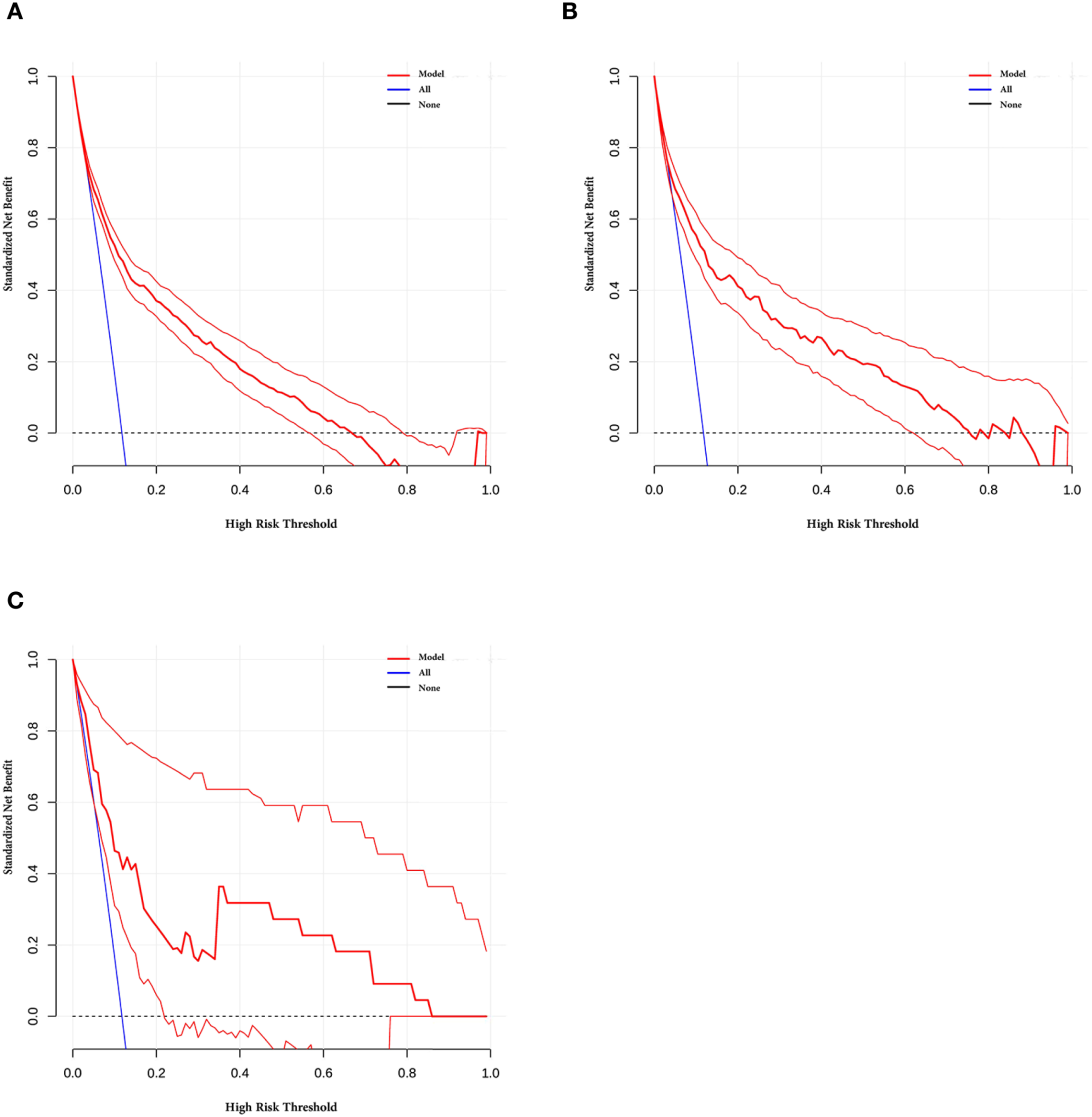
The Decision Curve Analysis (DCA) of the prediction model in the **(A)** training set, **(B)** internal validation set, and **(C)** external validation set.

The decision curve analysis (DCA) (**Figure 6**) shows that, when an appropriate risk threshold is chosen, this model provides optimal clinical decision-making benefits. In contrast, strategies of total intervention or no intervention did not offer better clinical net benefit. Analysis across all three study cohorts demonstrate that the prediction based on this model has greater clinical value compared to indiscriminate intervention or non-intervention strategies. Therefore, this multi-factor combined model holds promising potential for clinical decision-making in prostate cancer.

To facilitate clinical implementation, the optimal probability threshold identified by the Youden index is approximately 31% (0.31). At this threshold, the diagnostic performance was: training set (n = 1, 990): sensitivity 72.0%, specificity 76.6%; internal validation set (n = 854): sensitivity 73.9%, specificity 77.7%; external validation set (n = 281): sensitivity 71.6%, specificity 75.6%. Decision Curve Analysis demonstrated positive net clinical benefit for the nomogram across a wide range of threshold probabilities (approximately 10%–60%) compared to both “biopsy all” and “biopsy none” strategies. Among the 1, 959 benign patients in the training set, the 76.6% specificity would correctly identify approximately 1, 501 patients as low-risk, potentially avoiding these unnecessary biopsies.

## 4 Discussion

This multicenter retrospective study analyzed 3, 125 patients who underwent ultrasound-guided transrectal or transperineal prostate biopsy. By integrating routinely available clinical parameters - including prostate volume, tPSA, f/t PSA ratio and age - we developed and validated a nomogram-based predictive model. The established model demonstrates robust performance in stratifying prostate cancer risk, providing a noninvasive decision-support tool for primary care settings.

Age represents a well-established independent risk factor for prostate cancer, with multiple cohort studies demonstrating a positive correlation between advancing age and cancer risk, particularly for high-grade disease (14). A comprehensive analysis of 4, 298 cases revealed that men with prostate cancer exhibited significantly higher mean total PSA levels (8.58 ± 0.10 ng/mL) compared to those with benign disease (7.98 ± 0.07 ng/mL), while demonstrating significantly lower percent free PSA (13.62% ± 0.20% vs 17.58% ± 0.18%, p < 0.001) (15). This combination enhances diagnostic specificity, addressing the limitation of total PSA alone, which can be elevated in up to 27% of men with benign prostatic conditions (15). In this study, despite demonstrating statistical significance in univariate analysis (p < 0.05), fPSA was ultimately excluded from the predictive model due to identified multicollinearity issues. Total PSA (tPSA) has long been established as one of the most important independent predictors of prostate cancer (15, 16). The free-to-total PSA ratio (f/tPSA) enhances the specificity of PSA testing by identifying the characteristic decrease in free PSA fraction associated with prostate cancer (15, 17). These findings align with previous research demonstrating the differential diagnostic value of these PSA derivatives in prostate cancer risk stratification (15–17). Imaging parameters, particularly prostate volume measured by transrectal ultrasound, provide crucial additional predictive value in risk stratification. The European Randomized Study of Screening for Prostate Cancer (ERSPC) demonstrated that risk calculators incorporating prostate volume achieved superior discriminative performance with an area under the curve (AUC) of 0.79 for predicting prostate cancer, compared to 0.69 for PSA-only models (16). Given the established relationship between PSA levels and prostate volume, incorporation of volumetric data enables more accurate PSA interpretation and enhanced model precision (16). Validation studies in Asian populations have confirmed these findings, with nomograms incorporating age, PSA, and prostate volume demonstrating improved AUC from 79.7% (PSA alone) to 84.8% (combined model) (18).

The results of this study demonstrate that this multifactorial prediction model exhibited a significant advantage over individual parameters in clinical diagnosis, which is consistent with findings from previous investigations (19). The model not only improves the predictive accuracy for prostate cancer but also significantly reduces unnecessary biopsies, thereby alleviating the healthcare burden on patients (20, 21). This finding provides new insights into prostate cancer screening and early diagnosis (22, 23), particularly in primary healthcare settings, where such tools can be especially impactful. When compared to existing nomograms, the present study offers several distinctive advantages. First, unlike the ERSPC risk calculator (AUC 0.76–0.83 in external validation (24)) and PCPT risk calculator (25), which require digital rectal examination (DRE) and were primarily validated in North American/European Caucasian populations, our model uses only routine blood tests (tPSA, fPSA) and transrectal ultrasound (prostate volume)—parameters universally available in primary care settings. The MSKCC pre-biopsy nomogram requires PSA velocity kinetics data, limiting its utility at the first clinical visit. Second, the model was specifically developed and externally validated in a Chinese population (primary cohort n = 2, 844; external validation n = 281 from an independent institution), directly addressing the recognized gap in applicability of Western risk calculators to Asian patients (26). Third, the cohort encompasses both transrectal (764 cases, 26.9%) and transperineal (2, 080 cases, 73.1%) biopsy approaches, reflecting contemporary clinical practice trends in China. Fourth, in contrast to studies relying on specific biomarkers or high-cost equipment (27, 28, 36–38), this model uses only conventional clinical parameters, enabling widespread application in resource-limited environments.

Numerous large-scale cohort studies have previously developed various prostate cancer risk calculators to predict the probability of prostate cancer occurrence. Among these, the European Randomized Study of Screening for Prostate Cancer (ERSPC) risk tool and the Prostate Cancer Prevention Trial (PCPT) risk calculator (PCPTRC) are the most commonly used (24). Additionally, other continuously updated prediction models, such as the Prostate Biopsy Collaborative Group (PBCG) model, have been developed (25). Compared to the prediction model constructed in this study: In terms of predictive performance, the model developed here demonstrates comparable performance to some cohort-based results (in this study, the AUC was 0.816 (95% CI: 0.796–0.836) in the training set, 0.833 (95% CI: 0.802–0.862) in the internal validation set, and 0.776 (95% CI: 0.720–0.832) in the external validation set). In terms of clinical application, most existing risk calculators are primarily based on populations from North America or Europe. Although some studies have applied the PCPT risk score to assess prostate cancer risk in Chinese populations, they achieved only an AUC of 0.67, indicating the continued need for a predictive tool tailored to evaluate prostate cancer risk in Chinese individuals (26). The prediction model constructed in this study is based on data from 3, 125 cases across two tertiary medical institutions in China and has demonstrated stable and favorable predictive performance. This further highlights the potential clinical value of the prostate cancer prediction model developed in this research.

However, this study does have some limitations. Firstly, as a retrospective study, data collection and analysis may be subject to biases inherent in retrospective data, particularly in patient selection and data completeness. Secondly, while our model demonstrates good predictive performance, its generalizability to other regions requires further validation given our data were derived from two hospital-based cohorts in Southeastern China. Besides, our model was developed using a cohort of patients who underwent prostate biopsy based on clinical suspicion which may introduce bias. While this reflects real-world clinical practice, the model’s performance in asymptomatic or low-risk populations (e.g, PSA < 4 ng/mL) requires further validation. Finally, the heterogeneity in PSA assay methodologies (due to the retrospective multicenter design) constitutes a study limitation, as full standardization of measurements could not be achieved. However, this limitation reflects real-world clinical practice where PSA assays often vary between institutions, suggesting our findings may have broader generalizability despite the methodological constraint. Importantly, the present study did not incorporate multiparametric MRI (mpMRI) data, representing a key limitation. In centers where mpMRI is available, the EAU Guideline-recommended mpMRI-first pathway should be followed (29). Our nomogram is primarily intended for primary care settings where mpMRI is inaccessible, serving as a pre-biopsy risk stratification tool prior to referral decision (30). Additionally, our model was developed to predict “any prostate cancer” rather than exclusively “clinically significant PCa” (csPCa; ISUP ≥2). Incomplete ISUP grading in earlier records precluded a csPCa-specific primary analysis. Notably, csPCa (ISUP ≥2) accounted for 79.2% (701/885) of malignant cases in our cohort, indicating that the vast majority of detected cancers were clinically significant. Undoubtedly, future large-scale, multicenter prospective studies incorporating mpMRI and employing rigorous and standardized measurement protocols will be essential to further validate the model’s reliability and generalizability across diverse populations.

The results of this study provide a simple and cost-effective tool for the early screening and diagnosis of prostate cancer, which is particularly meaningful in reducing unnecessary biopsies and the healthcare burden associated with them. In regions with limited resources, traditional PSA testing may be prone to higher rates of misdiagnosis and unnecessary medical expenses (31). The nomogram model proposed in this study can offer clinicians more accurate decision support, optimizing biopsy and treatment strategies (32). Future research could explore how to further enhance the model’s predictive accuracy, for example, by incorporating emerging biomarkers or imaging data, which may improve the model’s performance. Additionally, integrating artificial intelligence technologies for personalized risk assessment could lead to innovative solutions for prostate cancer screening (33–35, 39–41).

## 5 Conclusion

This study developed and validated a nomogram model for prostate cancer risk assessment based on routinely available clinical parameters, using multicenter retrospective data from 3, 125 cases. The model demonstrated favorable predictive performance, calibration, and clinical net benefit in both internal and external validation, suggesting its potential utility in primary healthcare settings.

## Data Availability

The original contributions presented in the study are included in the article/Supplementary Material. Further inquiries can be directed to the corresponding authors.
